# An Inflammatory Cascade Leading to Hyperresistinemia in Humans

**DOI:** 10.1371/journal.pmed.0010045

**Published:** 2004-11-30

**Authors:** Michael Lehrke, Muredach P Reilly, Segan C Millington, Nayyar Iqbal, Daniel J Rader, Mitchell A Lazar

**Affiliations:** 1Divisions of Endocrinology, Diabetesand Metabolism, University of Pennsylvania School of Medicine, Philadelphia, PennsylvaniaUnited States of America; **2**Cardiology Department of Medicine, University of Pennsylvania School of MedicinePhiladelphia, PennsylvaniaUnited States of America; **3**Center for Experimental Therapeutics and Penn Diabetes Center, University of Pennsylvania School of MedicinePhiladelphia, PennsylvaniaUnited States of America; Albert Einstein College of MedicineUnited States of America

## Abstract

**Background:**

Obesity, the most common cause of insulin resistance, is increasingly recognized as a low-grade inflammatory state. Adipocyte-derived resistin is a circulating protein implicated in insulin resistance in rodents, but the role of human resistin is uncertain because it is produced largely by macrophages.

**Methods and Findings:**

The effect of endotoxin and cytokines on resistin gene and protein expression was studied in human primary blood monocytes differentiated into macrophages and in healthy human participants.

Inflammatory endotoxin induced resistin in primary human macrophages via a cascade involving the secretion of inflammatory cytokines that circulate at increased levels in individuals with obesity. Induction of resistin was attenuated by drugs with dual insulin-sensitizing and anti-inflammatory properties that converge on NF-κB. In human study participants, experimental endotoxemia, which produces an insulin-resistant state, causes a dramatic rise in circulating resistin levels. Moreover, in patients with type 2 diabetes, serum resistin levels are correlated with levels of soluble tumor necrosis factor α receptor, an inflammatory marker linked to obesity, insulin resistance, and atherosclerosis.

**Conclusions:**

Inflammation is a hyperresistinemic state in humans, and cytokine induction of resistin may contribute to insulin resistance in endotoxemia, obesity, and other inflammatory states.

## Abstract 2

Inflammatory stimuli affect resistin expression in human macrophages and raise serum resistin levels in healthy volunteers

## Introduction

Dietary and lifestyle changes during the last century have entailed an unprecedented epidemic of obesity and associated metabolic diseases, including type 2 diabetes and atherosclerosis [1]. Many individuals suffer simultaneously from more than one of these conditions, and epidemiological studies in humans, as well as studies in animal models, suggest that obesity-related insulin resistance is a common pathogenic feature [2]. Indeed, insulin resistance is the keystone of the “metabolic syndrome,” a major cardiovascular risk factor even in the absence of demonstrable glucose intolerance or diabetes [3]. Obesity and insulin resistance are strongly associated with systemic markers of inflammation, and, indeed, inflammation may contribute to insulin resistance [4]. Similarities and overlap between obesity and inflammatory states are emerging. Inflammatory cytokines such as tumor necrosis factor α (TNF α) and interleukin (IL)-6 are produced by adipocytes as well as by monocytes and macrophages, and they circulate at increased levels in individuals with obesity [5,6]. Moreover, bone-marrow-derived macrophages home in on adipose tissue in individuals with obesity [7,8], and adipocytes and macrophages may even be interconvertible [9]. Furthermore, inflammation is increasingly recognized as a major component and predictor of atherosclerotic vascular disease, a major clinical consequence of insulin resistance [10]. Hence, the interrelationships between obesity, insulin resistance, and atherosclerosis are of great scientific and clinical interest.

We originally identified and characterized resistin as a circulating mouse adipocyte gene product that is regulated by antidiabetic drugs [11]. In rodents, resistin is derived exclusively from adipocytes [11,12], circulates at increased levels in obese animals [11], and causes dysregulated hepatic glucose production, leading to insulin resistance [13,14]. A syntenic gene exists in humans, but is expressed at higher levels in monocytes and macrophages than in adipocytes [15,16], raising questions about the relationship between resistin and human metabolic disease. Recently, several studies have suggested that metabolic abnormalities are associated with polymorphisms in the human resistin gene [17,18]. Furthermore, several studies, though not all, have reported increased serum resistin levels in patients with obesity, insulin resistance, and/or type 2 diabetes [19,20,21,22,23,24,25,26]. However, the mechanism and importance of increased resistin levels in human metabolic disease are not known.

Here we show that the endotoxin lipopolysaccharide (LPS), a potent inflammatory stimulant, dramatically increases resistin production by inducing secretion of inflammatory cytokines such as TNFα. This increase in resistin production is blocked by both aspirin and rosiglitazone, drugs that have dual anti-inflammatory and insulin-sensitizing actions and have been shown to antagonize NF-κB. Indeed, activation of NF-κB is sufficient to induce resistin expression, and loss of NF-κB function abolishes LPS induction of resistin. Resistin serum levels are increased dramatically by endotoxemia in humans, and correlate with a marker of inflammation in patients with type 2 diabetes. Thus, systemic inflammation leads to increased resistin production and circulating levels in humans. The increased level of resistin in humans with obesity is likely an indirect result of elevated levels of inflammatory cytokines characteristic of states of increased adiposity. Hence, obesity and acute inflammation are both hyperresistinemic states associated with insulin resistance.

## Methods

### Differentiation of Primary Human Macrophages

Peripheral blood mononuclear cells were isolated from whole blood of healthy donors following apheresis and elutriation. Greater than 90% of these monocytes expressed CD14 and HLA-DR. Cells were plated in 24-well plates at a density of 10^6^ cells per well, allowed to adhere for 4 h, then washed with Dulbecco's Modified Eagles Medium and further cultured in 10% FBS in Dulbecco's Modified Eagles Medium supplemented with 5 ng/ml GM-CSF (Sigma, St. Louis, Missouri, United States) to promote macrophage differentiation. All experiments were performed after overnight equilibration with macrophage serum-free medium (GIBCO, San Diego, California, United States; Invitrogen, Carlsbad, California, United States) supplemented with 5 ng/ml GM-CSF. Cells were treated with LPS (Sigma), aspirin (Sigma), SN50, and/or control peptide (Biomol, Plymouth Meeting, Pennsylvania, United States), MG132, PD98059, SB20358 (Calbiochem, San Diego, California, United States), and TNFα (R&D Systems, Minneapolis, Minnesota, United States). Neutralizing antibodies to TNFα, IL-6, and anti-IL-1β, as well as control IgG, were obtained from R&D Systems. Adenovirus expressing activated IKK in pAD easy with GFP and control vector was a generous gift from Steven Shoelson.

### RNA Isolation and Quantification

RNA was isolated using RNeasy Mini Kit (Qiagen, Valencia, California, United States), then subjected to DNase digestion followed by reverse transcription (Invitrogen). mRNA transcripts were quantified by the dual-labeled fluorogenic probe method for real-time PCR, using a Prism 7900 thermal cycler and sequence detector (Applied Biosystems, Foster City, California, United States). Real-time PCR was performed using Taqman Universal Polymerase Master Mix (Applied Biosystems). The primers and probes used in the real-time PCR were the following: Sense-Resistin, 5′- AGCCATCAATGATAGGATCCA-3′; Antisense-Resistin, 5′- TCCAGGCCAATGCTGCTTAT-3′; Resistin Probe, 5′-Fam- AGGTCGCCGGCTCCCTAATATTTAGGG-TAMRA-3′; Sense human 36B4 sense, 5′- TCGTGGAAGTGACATCGTCTTT-3′; Antisense 36B4, 5′- CTGTCTTCCCTGGGCATCA-3′; and 36B4 Probe, 5′-FAM- TGGCAATCCCTGACGCACCG-TAMRA-3′.

Primer and probe for TNFα were obtained from Applied Biosystems. The cycle number at which the transcripts of the gene of interest were detectable (CT) was normalized to the cycle number of 36B4 detection, referred to as deltaCT. The fold change in expression of the gene of interest in the compound-treated group relative to that in the vehicle-treated group was expressed as 2^−deltadeltaCT^, in which deltadeltaCT equals the deltaCT of the compound-treated group minus the deltaCT of the chosen control group, which was normalized to 1.

### ELISA

Resistin concentrations, in cell media and human plasma, were assessed with a commercially available ELISA (Linco Research, St. Charles, Missouri, United States) and normalized to cell protein. The average correlation coefficient for standards using a four-parameter fit was 0.99. Intra-assay and inter-assay coefficients of variance were 4.7% and 9.1%, respectively. Direct comparison of standard curves generated by the Linco kit with those yielded by another commercially available resistin ELISA (Biovendor Laboratory Medicine, Brno, Czech Republic) yielded high correlation (rho = 0.99, *p <* 0.001), except that the Biovendor values were approximately 30% lower than those determined with the Linco assay. This appeared to be related to the standards used for calibration. Discrepant absolute values among different assays, including the Biovendor assay, were recently described by others [22]. Resistin levels in 40 plasma samples were measured using both Linco and Biovendor ELISA kits, with moderate correlation (rho = 0.66). Levels of soluble TNFα receptor 2 (sTNFR2) were measured using a commercially available immunoassay (R&D Systems). Intra-assay and inter-assay coefficients of variance were 5.1% and 9.8%, respectively.

### Human Endotoxemia Study

Healthy volunteers (*n* = 6, three male and three female), aged 18–45 y with BMI between 20 and 30 and on no medications, were studied. The University of Pennsylvania Institutional Review Board approved the study protocol, and all participants gave written informed consent. Following screening and exclusion of individuals with any clinical or laboratory abnormalities, participants were admitted to the General Clinical Research Center at the University of Pennsylvania for a 60 h stay. Serial blood samples were collected during the 24 h prior to and 24 h following the intravenous administration of human-research-grade endotoxin (obtained from National Institutes of Health Clinical Center, reference endotoxin [CCRE] [lots 1 and 2; National Institutes of Health Clinical Center PDS #67801]) at a dose of 3 ng/kg given at 6 AM. Plasma and whole blood RNA (PAX tube isolators, Qiagen) samples were isolated from blood, and stored under appropriate conditions for subsequent assays.

### Type 2 Diabetes Study

Participants with type 2 diabetes (*n* = 215, 167 male and 48 female), aged 35–75 y and free from clinical cardiovascular diseases, were recruited through the diabetes clinics at the University of Pennsylvania Medical Center and the Veterans Affairs Medical Center, Philadelphia, Pennsylvania, to an ongoing study of cardiovascular risk factors in type 2 diabetes. The sample was composed of 59% Caucasians and 35% African-Americans. All participants were evaluated at the University of Pennsylvania General Clinical Research Center in a fasting state at 8 AM. The University of Pennsylvania Institutional Review Board approved the study protocol, and all participants gave written informed consent. The patient population is described in more detail elsewhere [27].

### Statistical Methods

Data are reported as mean and standard error of the mean (SEM) for continuous variables. Because of baseline variation in cell populations between batches of primary human monocytes isolated from multiple donors, cell culture experiments were performed in triplicate and data from representative experiments are presented. For cell culture experiments with multiple treatments, analysis of variance (ANOVA) was used to test for differences in means across treatment groups. When significant global differences were found, post hoc *t*-tests were used to compare specific treatment groups to the control. Data from the human endotoxemia experiment were analyzed by repeated measures ANOVA. In the type 2 diabetes study, Spearman correlations of plasma levels of resistin with plasma sTNFR2 levels are presented.

## Results

### Induction of Resistin Gene and Protein Expression by Endotoxin Treatment of Human Macrophages

**Figure 1:**
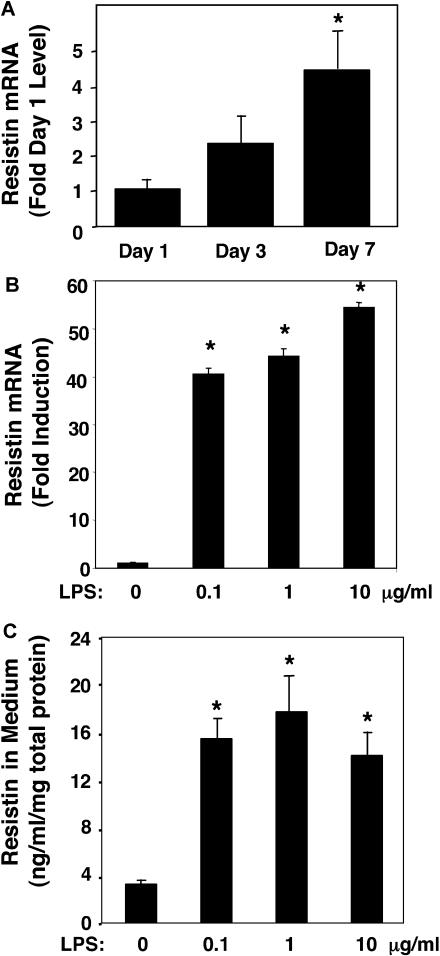
Induction of Resistin in Human Macrophages (A) Induction of resistin during human macrophage differentiation ex vivo. Expression of resistin on days 1, 3, and 7 following isolation and culture of human peripheral blood monocytes under macrophage differentiation conditions. Results shown are the mean (± SEM) of three separate experiments with triplicate samples. The ANOVA *F* statistic for change of resistin mRNA expression during differentiation was 7.06 (*p <* 0.01). *, *p <* 0.01 for post hoc *t*-tests. (B) Resistin mRNA is induced by endotoxin in primary human macrophage cultures. The ANOVA *F* statistic for change of resistin mRNA expression in response to increasing concentration of LPS (24 h treatment) was 423.57 (*p <* 0.001). *, *p <* 0.001 for post hoc *t*-tests. (C) Resistin protein secretion by human macrophages is induced by endotoxin. The ANOVA *F* statistic for change of resistin protein secretion in response to increasing concentration of LPS (24 h treatment) was 35.36 (*p <* 0.001). *, *p <* 0.001 for post hoc *t*-tests. For LPS dose response studies, shown in (B) and (C), results (mean ± SEM) of representative experiments, with triplicate samples, are presented. Similar results were obtained in two independent experiments.

The regulation of resistin expression was studied in primary cultures of human monocytic cells. Immediately upon plating of elutriated primary human monocytes, resistin gene expression was detectable but highly variable from experiment to experiment (data not shown). One day after plating, resistin gene expression remained detectable at low levels (Figure 1A). Subjection of the cells to a protocol leading to differentiation along the macrophage lineage led to a modest, time-dependent enhancement of resistin gene expression (Figure 1A). In agreement with a previous report [28], treatment of primary macrophages with the endotoxin LPS led to a dramatic, dose-responsive increase in resistin gene expression (Figure 1B). We also determined that this effect of LPS was paralleled by an increase in resistin protein secretion into the medium (Figure 1C). Of note, activated mouse peritoneal macrophages harvested after thioglycolate treatment did not express detectable levels of mouse resistin, even after treatment with LPS (data not shown).

### Endotoxin Induction of Resistin Is Delayed with Respect to TNFα

**Figure 2:**
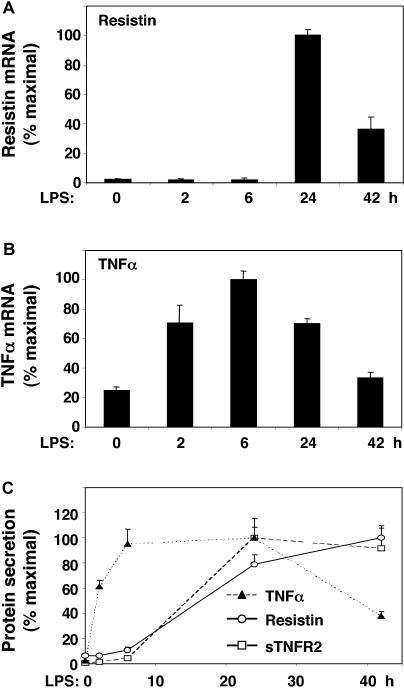
Endotoxin Induction of Resistin Occurs after Induction of TNFα Primary cultures of human macrophages were treated with LPS (1 μg/ml) for various times. (A) Time course of induction of resistin mRNA. The ANOVA *F* statistic for the change in resistin mRNA over time was 105.45 (*p <* 0.001). (B) Time course of induction of TNFα mRNA. The ANOVA *F* statistic was 34.57 (*p* < 0.001). (C) Time course of secretion of resistin, TNFα, and sTNFR2 into medium. ANOVA *F* statistics for the effect of LPS on resistin (66.51, *p <* 0.001), sTNFR2 (12.86, *p <* 0.001), and TNFα (20.48, *p <* 0.001) were highly significant. Maximal secreted protein levels were as follows: resistin, 21.9 ng/ml/mg; TNFα, 207.2 ng/ml/mg; and sTNFR2, 39.3 ng/ml/mg. Results of representative experiments with triplicate samples are expressed as mean (± SEM). Similar results were obtained in three independent experiments.

Induction of resistin gene expression by LPS exposure of human macrophages began between 6 and 24 h after treatment, with peak expression at 24 h (Figure 2A). This time course of resistin induction was delayed relative to induction of TNFα gene expression, which was detectable at 2 h and peaked 6 h after LPS exposure (Figure 2B). The secretion of TNFα followed a similar time course (Figure 2C). By contrast, secretion of resistin did not increase until much later, more closely following the pattern of the appearance of sTNFR2, a marker of TNFα action (Figure 2C) [29].

### Endotoxin Induction of Resistin Is Blocked by Immunoneutralization of Multiple Cytokines

**Figure 3:**
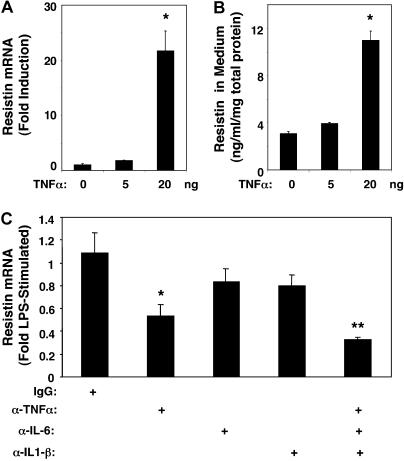
Endotoxin-Induced Cytokines Regulate Resistin Induction (A) TNFα induces production of resistin mRNA by primary human macrophages. The ANOVA *F* statistic for the effect of increasing TNFα concentrations on resistin was 23.81 (*p <* 0.001). *, *p <* 0.001 for post hoc *t-*tests. (B) TNFα induces resistin protein secretion by primary human macrophages. ANOVA *F* statistic for the effect of TNFα on resistin was 79.85 (*p <* 0.001). *, *p <* 0.005 for post hoc *t*-tests. Results of representative experiments with triplicate samples are expressed as the mean (± SEM). Similar results were obtained in two independent experiments. (C) LPS (1 μg/ml) induction of resistin is abrogated by antibody neutralization of cytokines (7.5 μg/ml per antibody). ANOVA *F* statistic for the effect of neutralizing antibodies on resistin was 3.08 (*p <* 0.05). *p*-Values for post hoc *t*-tests versus IgG: *, *p <* 0.05; **, *p <* 0.001. Results are expressed as the mean (± SEM) of three separate experiments with triplicate samples.

Resistin gene expression was also induced by TNFα treatment of primary human macrophages (Figure 3A) [28], and resistin secretion increased in parallel (Figure 3B). Since LPS induction of TNFα preceded the increase in resistin (see Figure 2C), we hypothesized that TNFα, or a similar cytokine produced early after LPS exposure, was responsible for the later induction of resistin. Indeed, neutralizing antibodies to TNFα markedly attenuated the increase in resistin gene expression (Figure 3C). LPS treatment also induces other cytokines, including IL-6 and IL-1β [30], and IL-6 induces resistin modestly (data not shown) [28]. Antibodies to IL-6 and IL-1β individually had minor effects on LPS stimulation of resistin (Figure 3C). However, the combination of antibodies to TNFα, IL-6, and IL-1β markedly attenuated LPS induction of resistin (Figure 3C). These data clearly show that resistin induction by endotoxin is mediated by a cascade in which the primary event is secretion of inflammatory cytokines that, in turn, induce resistin.

### Induction of Resistin Is Blocked by Anti-Inflammatory Insulin-Sensitizing Drugs That Target NF-κB

**Figure 4:**
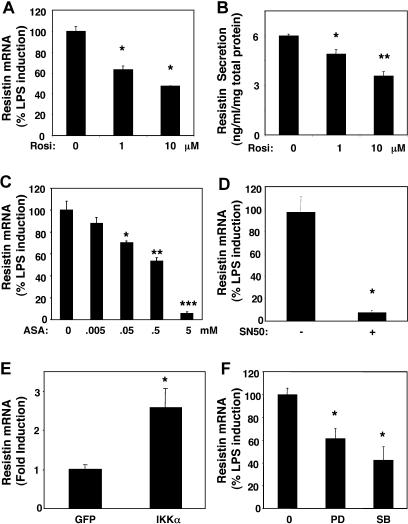
Inhibition of Resistin Induction by Anti-Inflammatory Insulin Sensitizers (A) Down-regulation of resistin mRNA by rosiglitazone. ANOVA F statistic for the effect Rosiglitazone on resistin expression was 62.52 (*p <* 0.001). *p* value for post hoc t-tests, is depicted in the Figure. *p < 0.005 versus control for post hoc *t*-tests. (B) Down-regulation of resistin protein secretion by human macrophages treated with rosiglitazone. The ANOVA *F* statistic for the effect of rosiglitazone on resistin protein secretion was 29.44 (*p <* 0.001). *p*-Values for post hoc *t*-tests versus control: *, *p <* 0.05; **, *p <* 0.001. Cells were pre-treated with rosiglitazone for 24 h and with LPS (1 μg/ml) and rosiglitazone for an additional 24 h. Results of representative experiments with triplicate samples are expressed as mean (± SEM). Similar results were obtained in three independent experiments. (C) Down-regulation of resistin gene expression by aspirin. The ANOVA *F* statistic for the effect of aspirin on resistin expression was 61.33 (*p <* 0.001). *p*-Values for post hoc *t*-tests versus no aspirin: *, *p <* 0.01; **, *p <* 0.001; ***, *p <* 0.0001. Cells were pre-treated with aspirin for 2 h and with LPS (1 μ g/ml) and aspirin for an additional 24 h. Results of representative experiments with triplicate samples are expressed as mean (± SEM). Similar results were obtained in two independent experiments. (D) Down-regulation of resistin gene expression by NF-κB inhibitor SN50. *, *p <* 0.001 versus control peptide by *t*-test. Cells were pre-treated with SN50 or control peptide at 100 ug/ml for 2 h, and with LPS (1 μg/ml) and SN50 or control peptide for an additional 24 h. Results are the expressed as the mean (± SEM) of two independent experiments performed in triplicate. (E) Induction of resistin by activation of NF-κB. *, *p <* 0.05 versus control virus by *t*-test. Cells were infected with adenovirus expressing activated IKK or control virus for 24 h. Results of representative experiments with triplicate samples are expressed as mean (± SEM). Similar results were obtained in two independent experiments. (F) Down-regulation of resistin gene expression by inhibitors of p38 and p42 MAPK. The ANOVA *F* statistic for the effect of the MAPK inhibitor on resistin expression was 11.54 (*p <* 0.005). *, *p <* 0.005 versus control for post hoc *t*-tests. Cells were pretreated with 50 μM PD98059 or 2.5 μM SB20358 for 2 h and with LPS (1 μg/ml) and PD98059 or SB20358 for an additional 24 h. Results are expressed as the mean (± SEM) of two independent experiments performed in triplicate.

Mouse resistin, produced exclusively by adipocytes, is down-regulated by antidiabetic thiazolidinediones, including rosiglitazone [11]. Consistent with an earlier report [16], rosiglitazone down-regulated resistin gene expression (Figure 4A) in LPS-stimulated human macrophages. Resistin protein secretion was also significantly reduced by rosiglitazone (Figure 4B). Hence, macrophage expression of resistin and its induction by LPS is species-specific, but down-regulation of resistin by thiazolidinedione occurs both in rodents and humans. Rosiglitazone has marked anti-inflammatory effects on macrophages [31]. This led us to examine the effect of aspirin, an anti-inflammatory compound that targets IκB kinase and has insulin-sensitizing effects [32]. Remarkably, aspirin dramatically decreased endotoxin-induced resistin expression in a dose-dependent manner (Figure 4C). Both aspirin (via IκB kinase) and rosiglitazone (via PPARγ) inhibit NF-κB [31,32], which is activated by LPS. Indeed, treatment of the macrophages with the proteasome inhibitor MG132, which prevents NF-κB activation [33], abrogated endotoxin-induced activation of resistin expression (data not shown). Moreover, treatment of the macrophages with SN50, a cell-permeable peptide that specifically prevents activation of NF-κB by inhibiting its nuclear translocation [34], nearly abolished endotoxin-induced activation of resistin expression (Figure 4D). Thus, activation of NF-κB is required for LPS induction of resistin in human macrophages. Furthermore, constitutive activation of NF-κB by adenoviral expression of activated IκB kinase was sufficient to induce resistin in primary human macrophages (Figure 4E). The magnitude of this activation was less than that caused by LPS, which is known to also activate MAP-kinase (MAPK). Indeed, inhibition of either p42 MAPK by PD98059, or p38 MAPK (using SB20358) partially blocked the induction of resistin by LPS (Figure 4F). Together these results show that NF-κB activation is necessary and sufficient for resistin induction by LPS, with MAPK activation increasing the magnitude of the response.

### LPS Robustly Increases Circulating Resistin Levels in Healthy Humans

**Figure 5:**
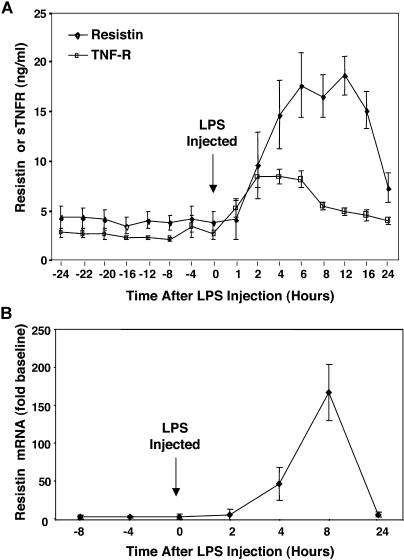
Endotoxin Dramatically Induces Plasma Resistin in Humans (A) Plasma resistin and sTNFR2 levels were measured serially in six healthy volunteers for 24 h before and after intravenous LPS (3 ng/kg) administration. The repeated measures ANOVA *F* statistics for the effect of LPS on plasma resistin (9.25, *p <* 0.001) and sTNFR2 (23.65, *p <* 0.001) were highly significant**.** (B) Mean resistin RNA expression in whole blood cells of healthy volunteers (*n* = 2) before and after treatment with LPS (3 ng/kg).

Next, we asked whether our findings from ex vivo studies of human macrophages would translate into in vivo observations in humans. Six healthy volunteers were injected with LPS, using a protocol similar to that shown to produce insulin resistance [35]. Baseline circulating resistin levels were approximately 4 ng/ml, and remained relatively constant for several hours prior to LPS infusion (Figure 5A). Remarkably, resistin levels rose dramatically because of endotoxemia, peaking 8–16 h after LPS administration (Figure 5A). The time course of hyperresistinemia paralleled the increase in circulating levels of sTNFR2, although the increase in resistin levels was more marked and sustained (Figure 5A). The increase in resistin protein levels correlated with increased resistin gene expression in peripheral blood mononuclear cells following systemic endotoxemia (Figure 5B).

### Circulating Resistin Levels Correlate with the Inflammatory Marker sTNFR2 in Patients with Type 2 Diabetes

**Figure 6:**
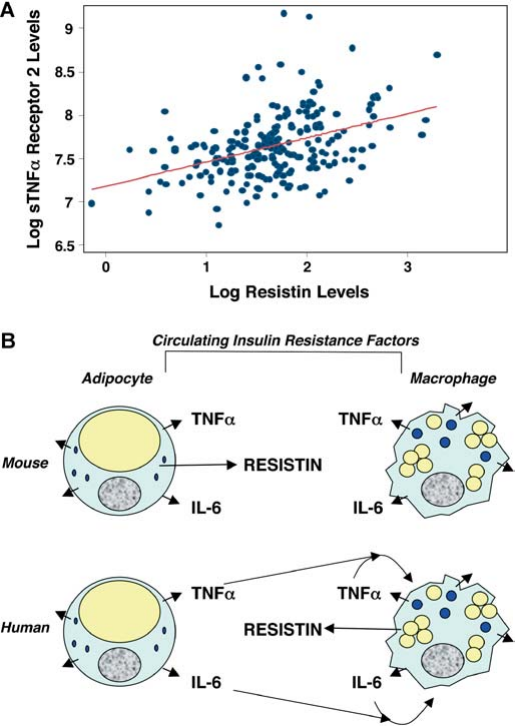
Plasma Resistin Levels Correlate with sTNFR2 Levels in Humans with Type 2 Diabetes (A) The correlation (Spearman coefficient rho = 0.38, *p <* 0.001) of plasma resistin and sTNFR2 levels in 215 humans with type 2 diabetes is presented. The line represents the linear regression fit between log-transformed plasma levels of resistin and sTNFR2. (B) Model to explain hyperresistinemia in mice and humans with obesity despite the species differences in the source of plasma resistin. Circulating inflammatory cytokines TNFα and IL-6 are depicted because of their role in resistin induction in human macrophages and their implied role in insulin resistance. Other cytokines and inflammatory markers may also contribute to insulin resistance and/or resistin induction.

Patients with type 2 diabetes and insulin resistance, many of whom are obese, have elevated levels of several inflammatory markers, including IL-6, TNFα, and sTNFR2 [36]. LPS administration has been shown to induce acute insulin resistance in humans [37]. Given that LPS infusion increased resistin levels, we measured resistin in a cohort of 215 patients with type 2 diabetes. Circulating resistin levels were significantly correlated with levels of sTNFR (Figure 6A). Thus, there is an association between resistin levels and systemic inflammation in patients with type 2 diabetes.

## Discussion

We have demonstrated that, in human macrophages, an inflammatory cascade with secretion of cytokines, including TNFα and IL-6, is sufficient and necessary for the induction of resistin. Insulin sensitizers that have anti-inflammatory properties, including a synthetic PPARγ agonist as well as aspirin, suppress macrophage resistin expression, as does direct inhibition of NF-κB. Experimental endotoxemia in healthy volunteers, based on the well-established gram-negative bacterial inflammatory response in humans [38,39,40], induces a dramatic elevation of circulating resistin levels. Hence, resistin gene and protein expression are increased by inflammatory stimuli both ex vivo and in vivo.

In rodents, resistin is produced exclusively by adipocytes, regulates normal glucose homeostasis, and causes insulin resistance at high circulating levels [11,13]. Translation of resistin's metabolic effects from rodents to humans has been problematic because peripheral blood mononuclear cells and macrophages appear to be a primary source of resistin in humans [15,16]. This species difference in primary locus of expression is yet another example of the close and functionally overlapping relationship between adipocytes and macrophages [41]. Numerous studies have reported that circulating resistin levels are increased in human obesity [20,25,26,41] and diabetes [19,20,23,42,43]. Our data suggest that, whereas hyperresistinemia in obese rodents derives directly from adipocytes, human resistin is indirectly regulated by the inflammatory internal milieu of obesity (Figure 6B). Indeed, obesity is associated with elevated levels of cytokines whose systemic administration leads to impaired glucose homeostasis [36,44,45], such as TNFα and IL-6, which we show here to mediate the inflammatory induction of human resistin. Thus, in both species, adipose tissue is an endocrine organ containing adipocytes as well as macrophages that regulates energy metabolism and glucose homeostasis through secretion of multiple factors, including inflammatory cytokines [46].

Clearly the relationship between obesity, inflammation, and resistin expression is complex, and needs to be systematically studied in larger and varied patient populations. Intriguingly, we found a strong correlation between plasma levels of resistin and sTNFR2, the soluble cleavage product of the activated TNFα receptor, in diabetic patients. A comparable correlation between resistin and sTNFR2 (*R* = 0.31, *p <* 0.001) was found in a cohort of 879 non-diabetic individuals, in whom resistin levels independently correlated with coronary atherosclerotic disease (M. P. Reilly, M. Lehrke, M. L. Wolfe, A. Rohatgi, M. A. Lazar, and D. J. Rader, unpublished data).

LPS binds to pathogen-associated-molecular-pattern innate immune receptors, such as CD14 and Toll-like receptor 4, activating signal cascades involving NF-κB and MAPK [47] and thereby inducing the transcription and secretion of early cytokines, including TNFα and IL-1 [48]. We have shown here that these early cytokines are responsible for secondary induction or enhancement of resistin expression in macrophages. Hyperresistinemia impairs glucose homeostasis in rodents [49,50], and inflammatory states are associated with insulin resistance [36], which may serve as a physiological attempt to increase the provision of glucose to the brain under stress conditions. Indeed, induction of acute inflammation by administration of LPS causes insulin resistance in humans [37], and here we have demonstrated the concomitant induction of resistin. Interestingly, the peak in TNFα and IL-6 levels after LPS administration to humans precedes a phase of prolonged insulin resistance that begins approximately 6 h after LPS administration [37], closely approximating the time course of resistin induction. Hence resistin is a potential mediator of insulin resistance in humans with acute inflammation. Moreover, obesity is associated with activation of innate immunity [6], including the inflammatory mediators that induce resistin. In this context it is intriguing that resistin levels are increased in obesity [25,26] and that insulin-sensitizing agents such as aspirin and rosiglitazone, with disparate primary molecular targets, antagonize resistin induction. Indeed, thiazolidinedione suppression of resistin levels has recently been correlated with hepatic insulin sensitization [43]. Future work will be needed to better understand the relationship between circulating resistin levels and the insulin resistance characteristic of inflammatory states, including obesity.

**Patient Summary**

**Why Was This Study Done?**

There is a very close connection between obesity and diabetes: diabetes is more common among obese people, and people with type 2 diabetes know that weight control is an essential part of their diabetes treatment. But the link between extra body fat and diabetes remains a puzzle. Recent experiments in mice suggested that a hormone called resistin could be the missing link. One reason is that resistin levels respond to a particular class of diabetes drugs called thiazolidinediones. But studies in humans found that mice and humans are quite different when it comes to resistin. One difference is that in mice resistin is produced by fat cells, but in humans it is produced by special immune cells called macrophages that are involved inflammation. Researchers are now studying what role—if any—resistin might have in humans with obesity and diabetes and are studying the similarities in the ways in which the body reacts to obesity and inflammation.

**What Did the Researchers Do?**

The researchers examined what happens to resistin levels when human macrophages or human patients are exposed to substances that trigger inflammation.

**What Did They Find?**

The substances that trigger inflammation caused higher resistin levels, but resistin levels were lowered again by thiazolidinediones.

**What Does This Mean?**

Because in mice higher resistin levels (produced by fat cells) are linked to diabetes, one possibility is that obesity in humans, by being similar to inflammation, causes immune cells to make lots of resistin and hence promotes diabetes that way.

**What Next?**

More research is necessary to confirm these findings and to find out how important resistin is as a link between obesity and diabetes, and how resistin promotes diabetes.

**Additional Information**

United States National Institute of Diabetes, Digestive, and Kidney Diseases (NIDDK) information on obesity: http://www.niddk.nih.gov/health/nutrit/pubs/unders.htm

NIDDK information on diabetes: http://diabetes.niddk.nih.gov/

International Diabetes Federation: http://www.idf.org/

## Abbreviations

ANOVA: analysis of variance
IL: interleukin
LPS: lipopolysaccharide
MAPK: MAP-kinase
SEM: standard error of the mean
sTNFR2: soluble tumor necrosis factor receptor 2
TNFα: tumor necrosis factor α
